# Supplementary material for: Clinically adaptable polymer enables simultaneous spatial analysis of colonic tissues and biofilms
Source: NPJ Biofilms Microbiomes. 2020 Sep 24;6:33. doi: 10.1038/s41522-020-00143-x (PMC7518420; doi:10.1038/s41522-020-00143-x)
Supplement: Supplementary file 1 — Supplementary Information [file 41522_2020_143_MOESM1_ESM.pdf]

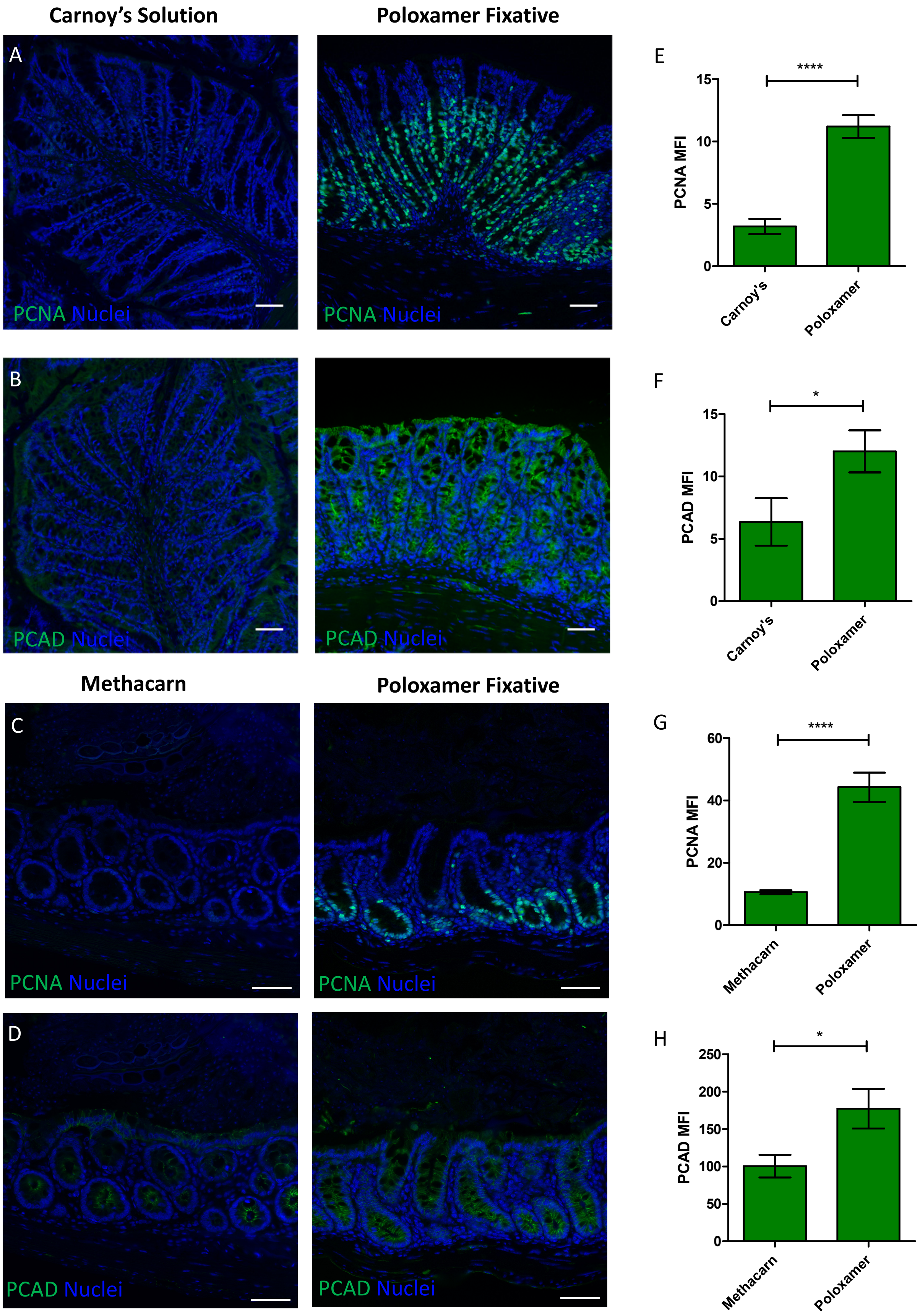

Supplementary Figure 1

Supplementary Figure 1: Poloxamer fixative preserves antibody staining

Comparison between Carnoy's and Poloxamer fixation of the murine colon for (A) PCNA, (B) pan-Cadherin (PCAD) antibody staining. (C-D) Comparison between Methacarn and Poloxamer fixation. (E-H) MFI-based image quantification of the corresponding comparisons from epithelial masks. Error bars are SEM from multiple fields of view from n=3 replicates. p-value *<0.05, ****<0.0001 by t-test. Scale bars = 50 µm.

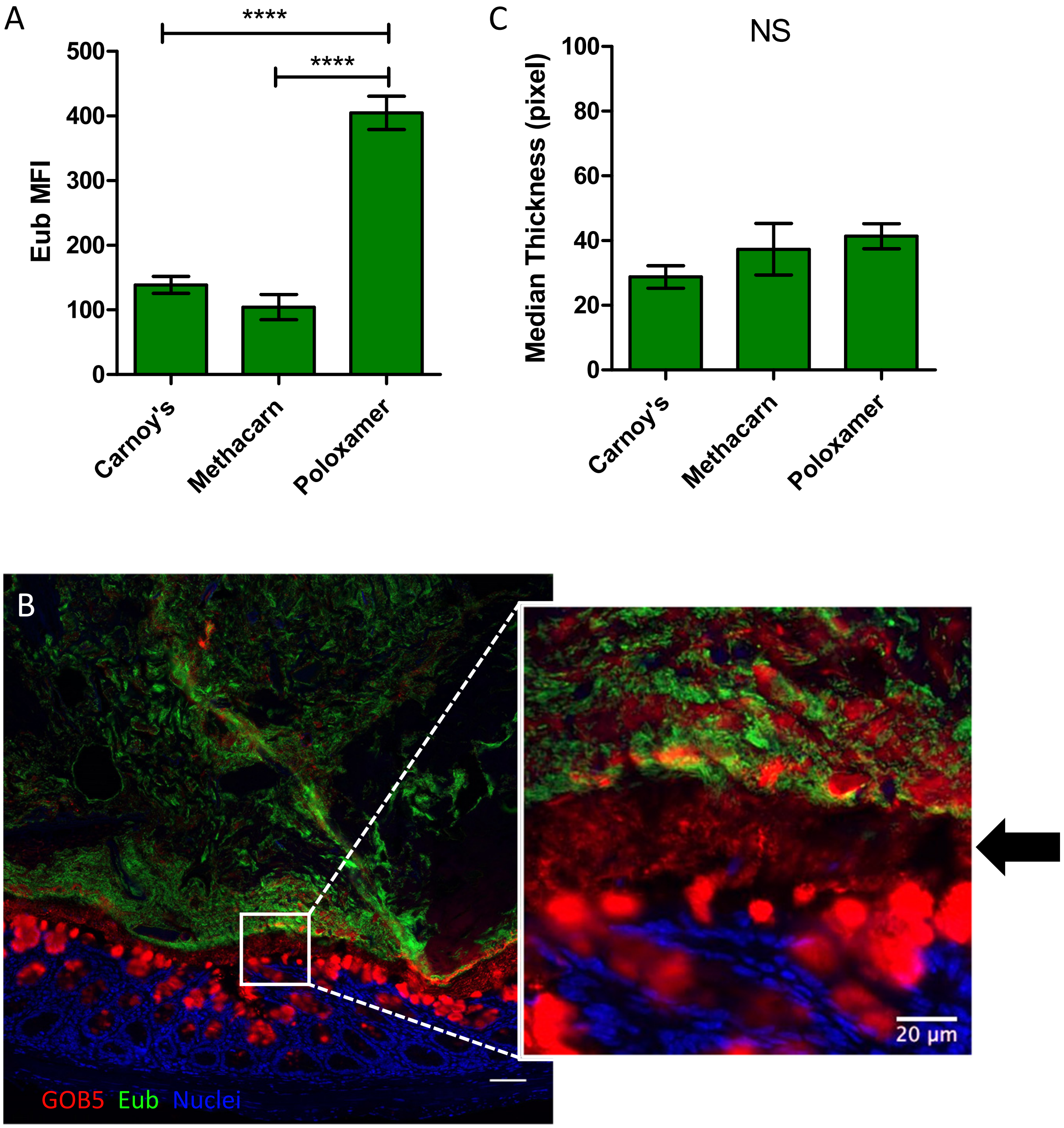

Supplementary Figure 2

Supplementary Figure 2: Closer examination of Poloxamer fixed mouse colon reveals intact inner mucus layer devoid of microbes. MFI-based image quantification of Eub stain intensity in outer mucus mask, comparing murine colon tissues prepared with (A) Carnoyl's solution, Methacarn, and Poloxamer fixative. (B) Mouse colon fixed using the Poloxamer fixative and stained with GOB5 (red) and Eub (green). Black arrow points to intact mucus layer. Scale bars = 50 µm and 20 µm (inset). (C) Median inner mucus layer thickness quantified by pixel measurements from images. Error bars are SEM from multiple fields of view from n=3 replicates. p-value NS not significant, ****<0.001 by ANOVA followed by Tukey post-test.

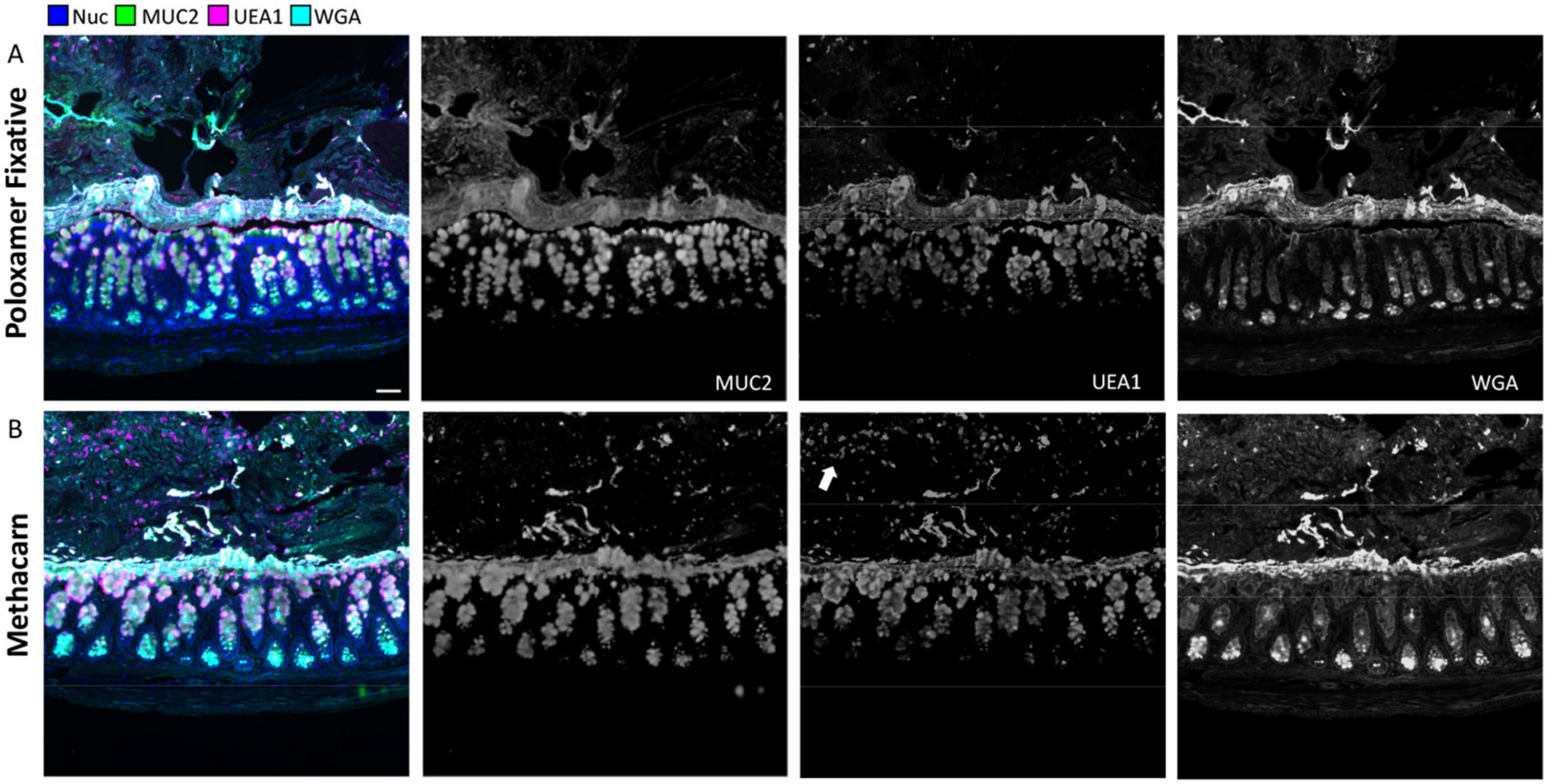

Supplementary Figure 3

Supplementary Figure 3: Lectin binds differently between Poloxamer fixative and Methacarn. MxIF of nuclei (blue), MUC2 (green), UEA1 (magenta), and WGA (cyan). Combined and individual channels to visualize each stain with white arrows pointing to non-specific, non-mucus cell staining in Methacarn. (A) Poloxamer fixative, (B) Methacarn. Scale bars = 50 μm.

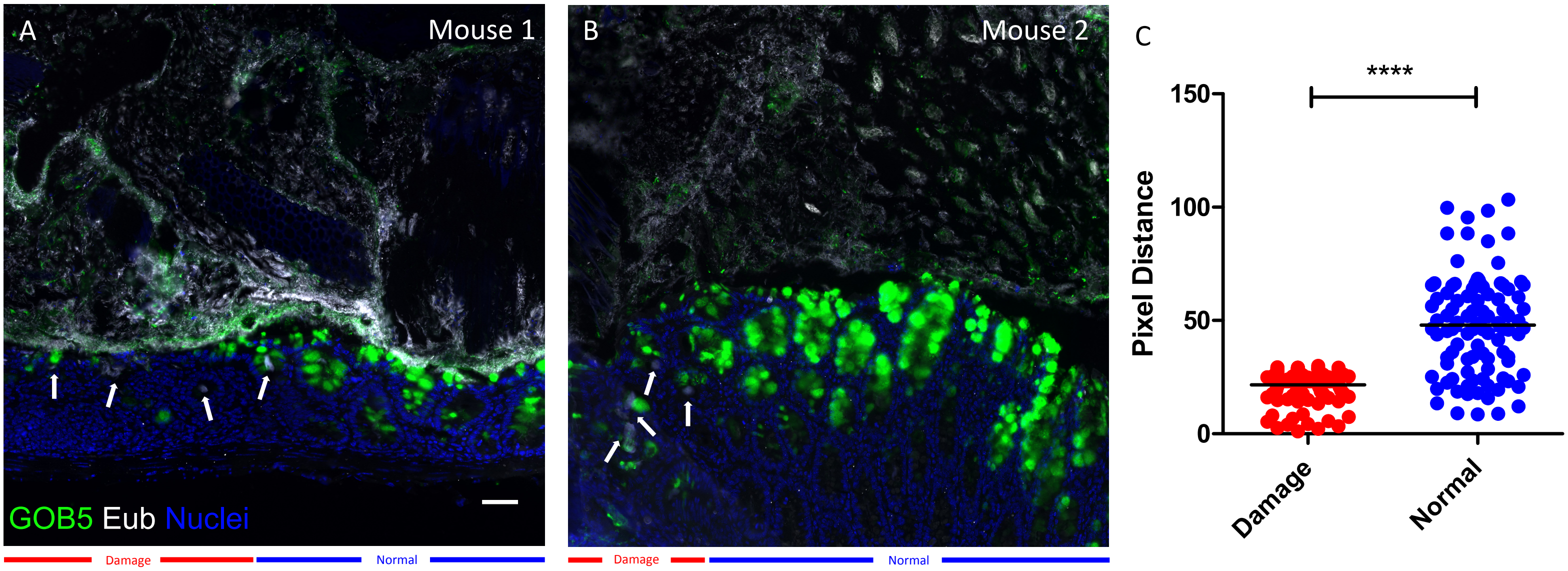

Supplementary Figure 4

Supplementary Figure 4: DSS damage results in a reduced inner mucus layer with Eub probe signal in crypts. (A-B) Representative IF of the colonic mucosa of n=2 mice treated with DSS. Damaged (red) and adjacent normal (blue) regions are noted. White arrows point to crypt with Eub probe signal in the damaged regions. Scale bars = 50 µm. (C) Median inner mucus layer thickness quantified by pixel measurements from images of mouse 2. Individual data points are single, shortest distances between the Eub probe and the epithelium. p-value ****<0.001 by t-test.

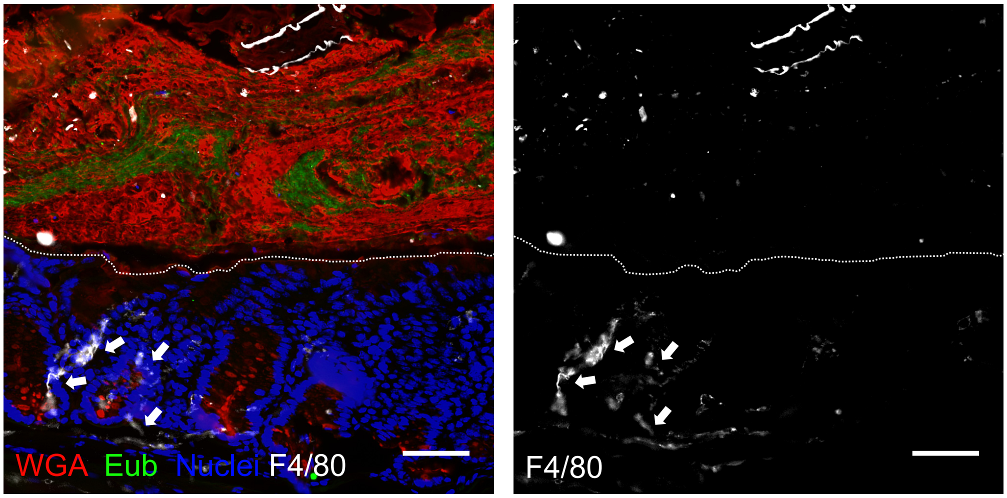

Supplementary Figure 5

Supplementary Figure 5: F4/80+ cells differentially infiltrate into the mouse colonic mucosa. Iterative FISH and IF of Poloxamer-fixed colonic tissues from germ-free mice colonized with the human biofilm slurry, with universal bacterial probe (Eub, green), nuclei (blue), GOB5 (green), lectin (WGA, red), and F4/80 (white). F4/80 channel to visualize macrophages displayed separately. White dotted lines represent epithelial borders. Scale bars = 50 µm.

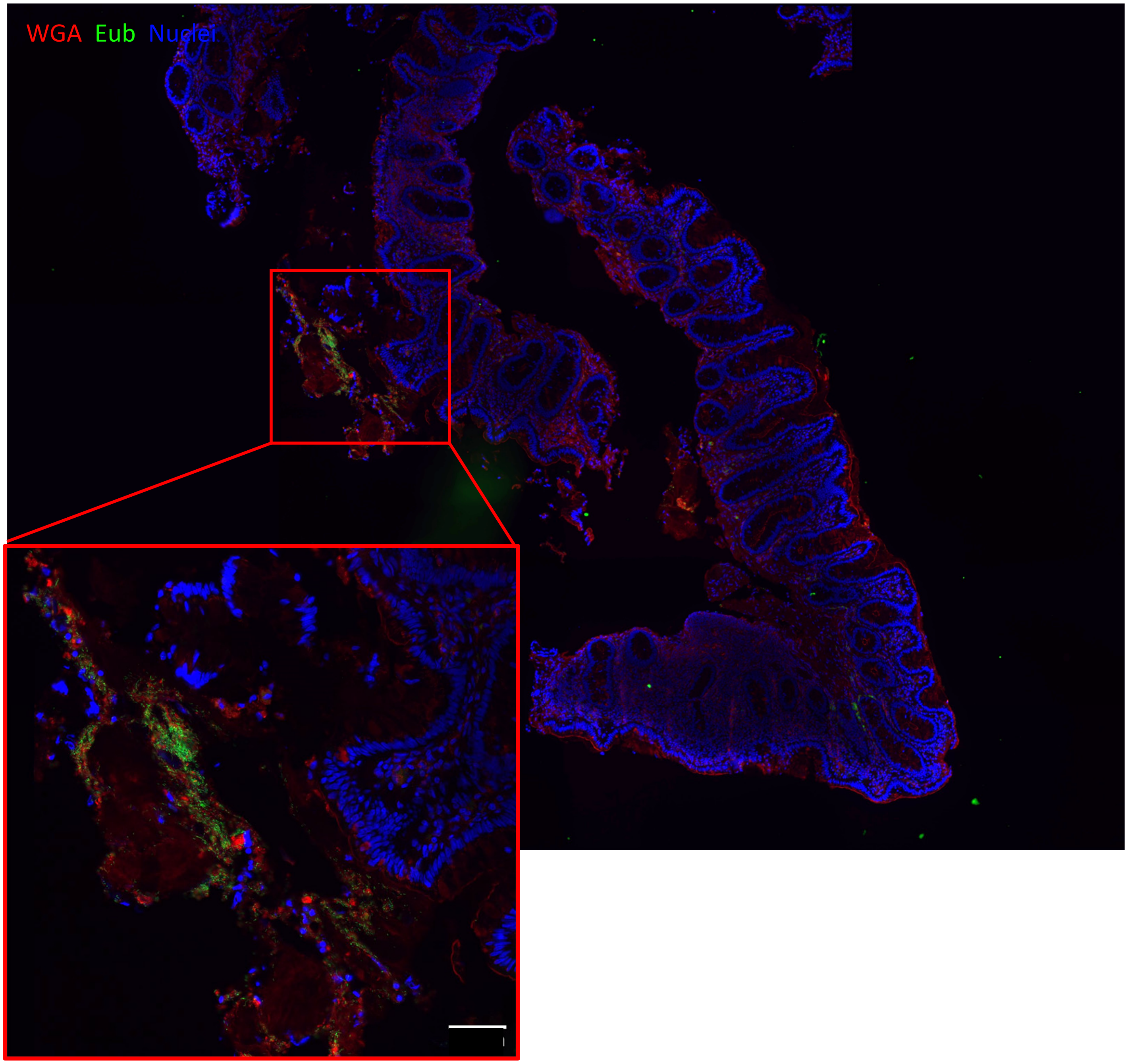

Supplementary Figure 6

Supplementary Figure 6: Normal human biopsy in fixed Poloxamer fixative reveals preserved architecture of the colonic mucosa. Scan of a human colon biopsy stained using WGA (red) for mucus and Eub (green) for bacteria. Scale bar = 50 μm.
